# Supplementary material for: The language of opinion change on social media under the lens of communicative action
Source: Sci Rep. 2022 Oct 26;12:17920. doi: 10.1038/s41598-022-21720-4 (PMC9605949; doi:10.1038/s41598-022-21720-4)
Supplement: Supplementary file 1 — Supplementary Information. [file 41598_2022_21720_MOESM1_ESM.pdf]

Supplementary Information

Corrado Monti[1], Luca Maria Aiello[2,3,*], Gianmarco De Francisci Morales[1], and Francesco Bonchi[1,4]

[1]CENTAI, Torino, Italy
[2]IT University of Copenhagen, Copenhagen, Denmark
[3]Pioneer Centre for AI, Copenhagen, Denmark
[4]Eurecat, Barcelona, Spain

Sociopolitical topic classification

In order to extract our data set of posts with a sociopolitical topic from `r/ChangeMyView`, we build a supervised classifier. The development and training of such a classifier is described in Section 5.1 in *Materials & Methods*. Here, we report further information on the quality of this classifier.

First, we build a test set with the same procedure we used for the training data of the classifier; that is, we aggregate posts from different subreddits after we categorize each subreddit as sociopolitical or not (see Section 5.1 for details). On this test set, we obtain an average F1 score of 89.5%. However, since the activity on Reddit varies significantly in the nine years we consider, we further investigate whether the performance of this classifier changes over time. Table SI1 reports the F1 score as measured on the posts from each year. We find that the quality in classification presents very limited variance over different years (i.e., within ± 2.5 p.p. from the global F1). Table SI2 reports additional metrics for this classifier.

Second, we build a validation set by selecting a random sample of `r/ChangeMyView` posts that we categorize as sociopolitical or not, according to the definition given by Moy and Gastil [16] (see *Research Design* section, page 2). Table SI3 reports a random excerpt of this validation data set, which provides concrete examples of the "sociopolitical" category. On this validation set, the classifier obtains an F1-score of 82% when considering the posts where text is present in the body of the post. Table SI2 also reports additional metrics on this data set.

Data

We apply the classifier in order to find all `r/ChangeMyView` posts with sociopolitical topic. Here, we further characterize the `r/ChangeMyView` data set gathered this way. Figure SI1a shows the number of posts per month over the time span of nine years considered in our analysis. Unlike the number of posts, which fluctuates over time, the fraction of posts that are sociopolitical is rather stable, suggesting that the discussion is not dominated by any event in particular. Figure SI1b reports the distribution of the number of posts per author in this set.

Finally, in our analysis we focus on the distinction between comments that received a Δ from the original author—which indicates the author admits to have changed their view after reading such comment—and those comments that did not receive one. Of the 3 690 687 comments, 38 165 were awarded a Δ. As an additional control group, we find 504 550 comments that did receive an answer from the original poster, and therefore received their attention, but did not obtain a Δ: this is further evidence that the original author did not consider such comments view-changing. We report the distribution of the number of posts answered by the original author with a given number of comments in each of these two categories (with and without Δ) per post in Figure SI1c (posts that have comments both with Δ and without count towards both distributions).

Social dimensions

Figure SI2 shows the probability distribution of comment length across dimensions. The typical length of messages may vary considerably across dimensions; for example, comments conveying status tend to be much shorter that knowledge-exchange comments.

Table SI1. Classification performance of our sociopolitical classifier on the test set of Reddit posts over the years. In italic, years for which there are no posts in `r/ChangeMyView`.

**Year**	*2011*	*2012*	2013	2014	2015	2016	2017	2018	2019
**F1-score**	*0.87*	*0.89*	0.89	0.89	0.90	0.92	0.91	0.90	0.89

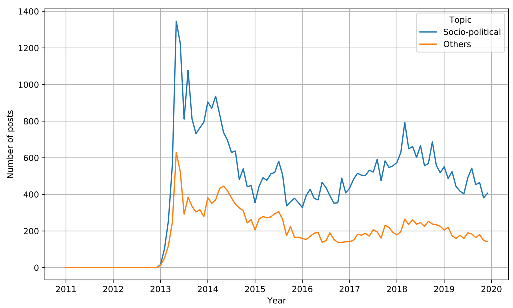

(a) Number of sociopolitical and not-sociopolitical posts on `r/ChangeMyView` per month over time, as categorized by our supervised classifier.

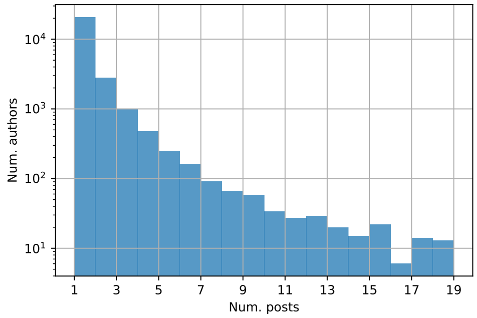

(b) Distribution of the number of posts per author in our set of sociopolitical ChangeMyView posts.

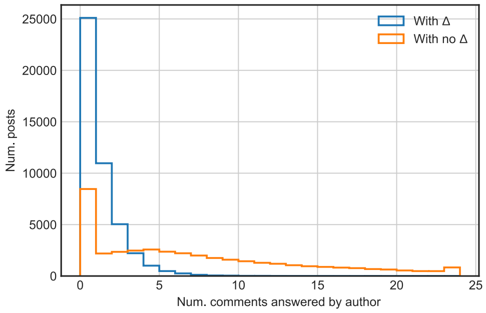

(c) Distribution of the number of posts with a given number of comments for each category (with Δ and with no Δ), in our set of sociopolitical ChangeMyView posts.

Figure SI1. Descriptive statistics of the sociopolitical posts and comments in `r/ChangeMyView`.

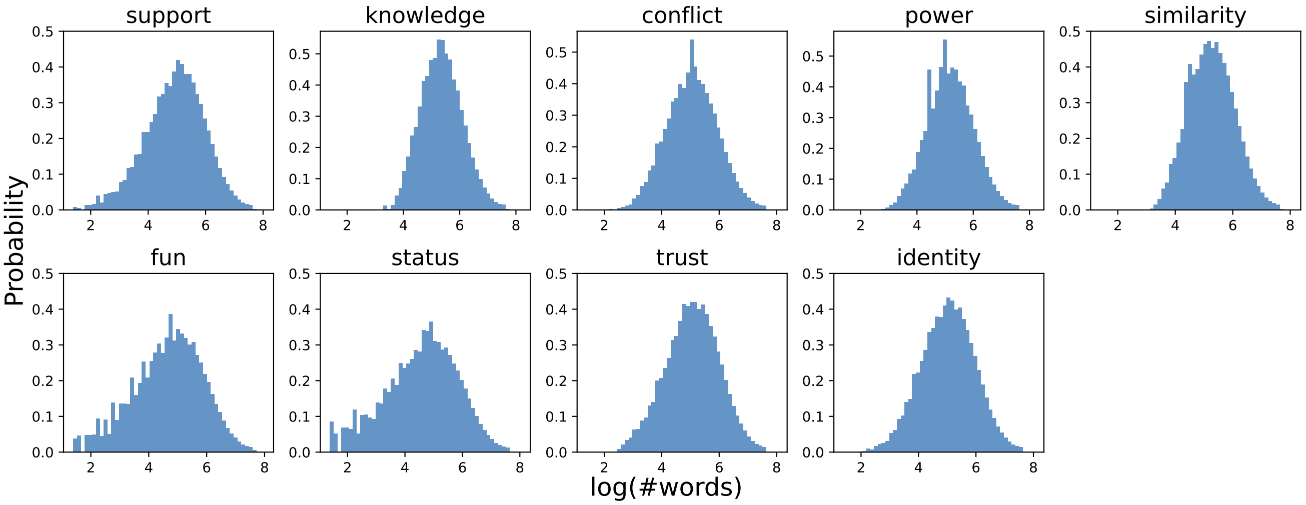

Figure SI2. Distribution of (log) number of words in comments with dimension d.

Figure SI3 shows the fraction of comments with dimension d among all the comments with a given range of length. We consider five length classes, corresponding to the quintiles of the length distribution. The probability of finding a dimension in a comment increases linearly with the comment length.

Figure SI4 shows the cross-correlations between the dimension scores, for all the dimension pairs (plus sentiment scores and comment length, measured in number of words). On the left, we report correlations computed on the original values $s_d(m)$. On the right, we report correlations computed on the weight-discounted values $d(m)$. The weight-discounting reduces the cross-correlations considerably.

Opinion change

Table SI4 shows the results of a logistic regression to predict whether a comment got a Δ by using (i) all the dimensions including fun, and (ii) with sentiment scores in addition to all the dimensions. The social dimension of *Fun* is not significant, we therefore remove it from the other regression models.

Table SI5 repeats the same analysis from Table 2 but including information about the length of the message. We do so by including as an independent variable the quantity $Z(\log l)$, where l is the length of the message and Z is a Z-score standardization. We apply a logarithmic scaling since the length of a message is broadly distributed. Note that this regression model is spurious, since there is an interdependence between the length of a message and the social dimensions conveyed by it. Namely, it usually takes more words to express some social dimensions rather than others. For example, to express *knowledge*, one typically requires to use a relatively large number of words to articulate an argument, whereas *status* can be conveyed effectively with just a few words of admiration.

Nonetheless, these results show that many of our findings are robust: in particular, all of the dimensions are highly significant (as in Table 2).

Figure SI5 shows the odds ratios calculated considering only comments that got a reply from the OP. Figure SI5a shows the the odds ratios of a social dimension being conveyed by comments with Δ compared to comments with no Δ. Figure SI5b shows the the odds ratios of a social dimension being conveyed by posts for which aΔ was awarded, compared to posts whose authors did not give any Δ. Figure Figure SI5c shows a matrix of interaction between the intent of the poster and that of the commenter. The results are qualitatively very similar to those obtained when considering all the comments, including those that got no reply from the OP.

Table SI2. Classification performance of the sociopolitical classifier on the automatically-built test set and on the human-evaluated validation set.

**Measure**	*F1-score*	*Precision*	*Recall*	*Accuracy*
**Test set**	*0.90*	*0.91*	*0.88*	*0.90*
**Validation set**	*0.82*	*0.76*	*0.88*	*0.77*

Table SI3. Examples from the validation set of ChangeMyView posts, hand-categorized as sociopolitical or not, according to our definition.

Sociopolitical	Title
Yes	CMV: The Second Amendment can no longer serve its intended purpose.
Yes	I believe in the event of a major catastrophic natural disaster, the government should let its citizens enjoy blissful ignorance until the last moment. CMV.
Yes	Fennisists and the metoo movement have caused more damage than good and are partially responsible for the rise of redpillers/mgtow
No	CMV: In terms of overall health, one 12 oz Coca Cola per day is worse than one cigarette per day.
Yes	CMV: Men should have a form of Abortion
Yes	I do not condone abortion in any capacity. CMV
Yes	I don't believe it is my responsibility to censor my actions or language to keep from offending others. CMV
Yes	CMV: Theism and Atheism are both invalid arguments.
No	CMV: Life is pointless
No	CMV: Subreddits that disable downvoting should not be eligible to make the front page.
Yes	CMV: Inciting Violence Should Be Protected Under Free Speech
No	CMV: I believe OJ Simpson , by himself, committed the murders of Ron Goldman and Nicole Brown Simpson.
Yes	CMV: America is not the greatest country in the world
Yes	CMV: I don't think anyone should have to pay child support. Ever.
Yes	CMV: Rewarding children for trying and participating (and winning) is the correct model; those who suggest just awarding the winners are wrong
No	I believe the artificial fluoridation of water is safe. CMV.
Yes	CMV: Many white people's social justice advocacy is self-serving and insincere.
No	CMV: The Lottery is not real, and the winners are paid actors.
Yes	CMV: Despite intuition to the contrary, I cannot see why a transracial person should not be accepted the way a transgender person is.
Yes	CMV: One is able to criticize Islamic texts without being labelled racist
No	CMV: /r/NoFap is bullshit
No	I think Zombies are extremely overused and Dinosaurs need to make a comeback. CMV

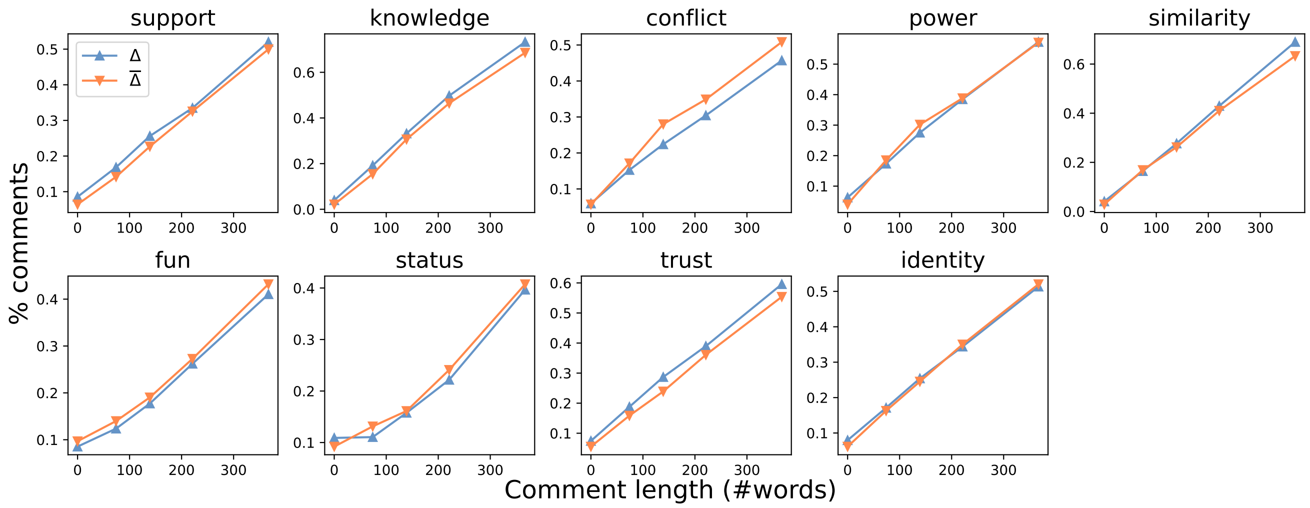

Figure SI3. Fraction of comments of a given length that have dimension d, separately for comments with and without Δ.

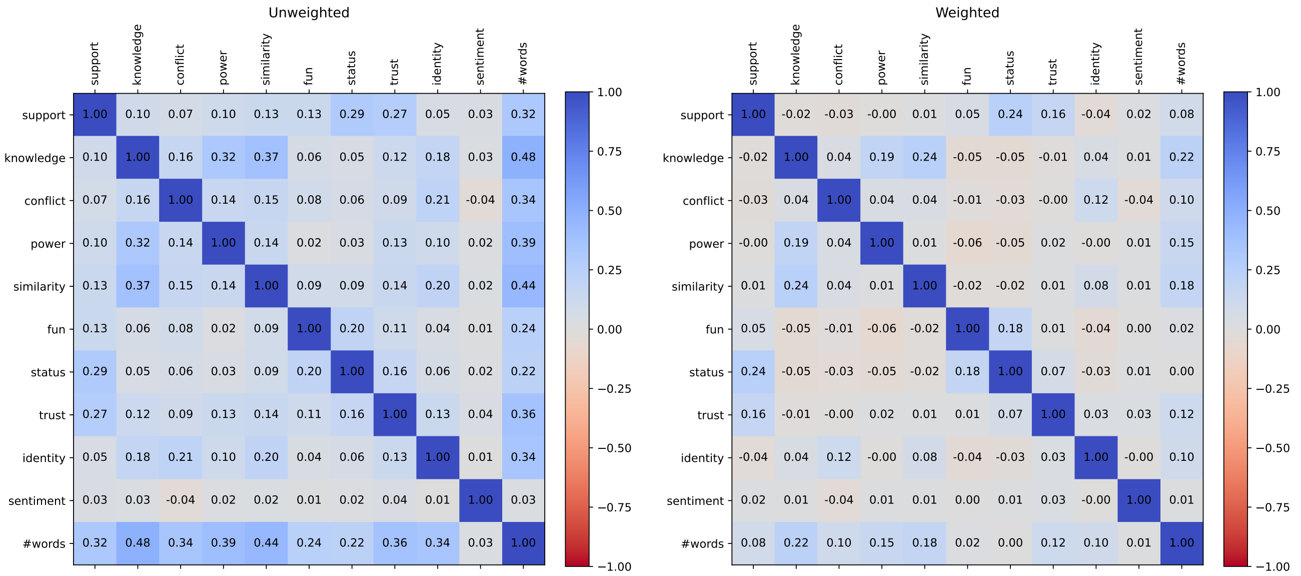

Figure SI4. Correlation matrix between variables before (left) and after (right) weight discounting.

Tables SI6, SI7, and SI8, report results of experiments we conducted as robustness checks.

Table SI6 reports the results of the regression model fit on a balanced data set obtained by undersampling negative examples. The results are very similar to the those reported in Table 2 in the main manuscript. The predictive power is slightly improved, simply because we remove the added difficulty of class imbalance and additional noise provided by the negative examples.

Table SI7 reports the results of a regression model fit on a randomized dataset that we obtained by randomly shuffling the social dimensions associated to each example, so that the association between opinion change and social dimensions is disrupted. In this experiment, no social dimension appears as significant, as one would expect.

Last, Table SI8 reports the results of the regression that we obtain when considering a more conservative threshold of the sociopolitical classifier (i.e., moving the threshold from 0.50 to 0.75). Such a change does not affect any of the significance levels that we record in the main regression model—most coefficients do not vary by more than 0.01 compared to those presented in Table 2 in the main manuscript. Also the variation in the Pseudo-R^2 is minimal. We can therefore conclude that, even if the accuracy of our sociopolitical classifier is not perfect, our results seem robust to minor classification errors.

Homophily and opinion change. Figure SI6a shows the likelihood that different dimensions are present in comments that are exchanged by two users with different profiles. Figure SI6a (left) shows the odds ratios of a dimension being present in a comment when the commenter and the author of the commented post participated in some political subreddits, but do not have one in common. Figure SI6a (right) shows the odds rations of a dimension being present in a comment when the commenter and the poster belong to different ideologies (left-wing vs. right-wing). Comments flowing between users with different profiles are more likely to convey power and conflict and less likely to contain support or status. Also, knowledge-rich discussion happens between people who have different stances.

Figure SI6b shows the result of a logistic regression model that includes as independent variables the social dimensions of the comment, the political side of the commenter, and the political side of the poster. The dependent variable is, as in Table 2, whether the comment is marked with a Δ or not (that is, if it is labelled as opinion-changing). We aggregate the resulting coefficients in order to obtain odds ratios for all of the nine possible configurations of these variables. Then, we normalize such odds ratios with respect to the interaction between two individual with no detected political side. From these results, we can observe:

1. heterophily: individuals are more likely to change their opinion when confronted by an individual of the opposite political side;

2. asymmetry between left-wing and right-wing individuals: a left-wing individual is more likely to receive a Δ from a right-wing one than vice versa.

Table SI4. Odds ratios obtained by a logistic regression that considers all social dimensions (including the non-significant *fun*), with and without traditional sentiment scores. We indicate with asterisks the statistically significant correlations (with one, two, or three asterisks corresponding to $p < 0.05$, $p < 0.01$ and $p < 0.001$ respectively). P-values are corrected according to the Benjamini-Hochberg procedure, to reduce the chance of spurious correlation emerging because of the high number of factors we consider.

Adj. Pseudo-R^2	0.01520	0.01643
Intercept	0.010***	0.010***
Support	1.111***	1.096***
Knowledge	1.226***	1.217***
Conflict	1.029***	1.024***
Power	1.099***	1.097***
Similarity	1.118***	1.110***
Fun	0.999	0.993
Status	0.935***	0.931***
Trust	1.155***	1.143***
Identity	1.088***	1.085***
Sentiment Neg.		1.056***
Sentiment Pos.		1.111***

Table SI5. Odds ratios obtained by logistic regression when considering also the length of the comment. Each column corresponds to a model with a specific set of variables. A description of each variable is given in Table 3. We indicate with asterisks the statistically significant correlations (with one, two, or three asterisks corresponding to $p < 0.05$, $p < 0.01$ and $p < 0.001$ respectively). P-values are corrected according to the Benjamini-Hochberg procedure, to reduce the chance of spurious correlation emerging because of the high number of factors we consider.

	A	B	C	D	E	F	G	H
Adj. Pseudo-R^2	0.06898	0.07054	0.07479	0.07283	0.07042	0.07193	0.07615	0.07421
Intercept	0.007***	0.007***	0.007***	0.007***	0.007***	0.007***	0.007***	0.007***
Support	....	....	....	....	1.033***	1.032***	1.034***	1.033***
Knowledge	....	....	....	....	1.066***	1.065***	1.066***	1.065***
Conflict	....	....	....	....	0.925***	0.927***	0.926***	0.926***
Power	....	....	....	....	0.974***	0.976***	0.973***	0.974***
Similarity	....	....	....	....	0.988*	0.986**	0.989*	0.988*
Status	....	....	....	....	0.916***	0.915***	0.923***	0.920***
Trust	....	....	....	....	1.051***	1.050***	1.050***	1.050***
Identity	....	....	....	....	0.999	0.999	0.999	0.999
Sentiment Pos.	1.054***	1.052***	1.056***	1.054***	1.043***	1.042***	1.044***	1.042***
Sentiment Neg.	0.979***	0.981**	0.980***	0.980***	0.996	0.998	0.997	0.997
Comment Left-Wing	....	0.984	....	....	....	0.983	....	....
Comment Right-Wing	....	0.772***	....	....	....	0.776***	....	....
Post Left-Wing	....	0.878***	....	....	....	0.880***	....	....
Post Right-Wing	....	0.847***	....	....	....	0.846***	....	....
Both Polarized	....	....	....	0.332***	....	....	....	0.334***
Both Polarized & Diff. Side	....	....	....	2.345***	....	....	....	2.338***
Diff. Side	....	....	....	1.017	....	....	....	1.017
Shared Group	....	....	0.297***	....	....	....	0.299***	....
Log Length	2.929***	2.929***	2.905***	2.914***	2.904***	2.906***	2.879***	2.890***

Table SI6. Odds ratios obtained by logistic regression with a balanced data set.

	A	B	C	D	E	F	G	H
Adj. Pseudo-R^2	0.00658	0.00938	0.01555	0.02031	0.03804	0.04065	0.04644	0.05093
Intercept	0.981**	1.064***	1.030***	1.053***	0.902***	0.978*	0.946***	0.967***
Support	....	....	....	....	1.126***	1.125***	1.128***	1.128***
Knowledge	....	....	....	....	1.272***	1.270***	1.269***	1.269***
Conflict	....	....	....	....	1.036***	1.038***	1.037***	1.036***
Power	....	....	....	....	1.138***	1.141***	1.139***	1.136***
Similarity	....	....	....	....	1.152***	1.149***	1.151***	1.152***
Status	....	....	....	....	0.920***	0.919***	0.924***	0.927***
Trust	....	....	....	....	1.177***	1.177***	1.177***	1.178***
Identity	....	....	....	....	1.102***	1.103***	1.104***	1.104***
Sentiment Pos.	1.211***	1.208***	1.211***	1.214***	1.136***	1.134***	1.135***	1.138***
Sentiment Neg.	1.095***	1.097***	1.097***	1.096***	1.071***	1.072***	1.072***	1.072***
Comment Left-Wing	....	1.016	....	....	....	1.013	....	....
Comment Right-Wing	....	0.788***	....	....	....	0.788***	....	....
Post Left-Wing	....	0.884***	....	....	....	0.890***	....	....
Post Right-Wing	....	0.835***	....	....	....	0.836***	....	....
Both Polarized	....	....	0.329***	....	....	....	0.332***	....
Both Polarized & Diff. Side	....	....	2.565***	....	....	....	2.540***	....
Diff. Side	....	....	1.021	....	....	....	1.025	....
Shared Group	....	....	....	0.294***	....	....	....	0.297***

Table SI7. Odds ratios obtained by logistic regression with randomized social dimensions.

	A	B	C	D	E	F	G	H
Adj. Pseudo-R^2	0.00305	0.00446	0.00720	0.00935	0.00303	0.00444	0.00718	0.00933
Intercept	0.010***	0.011***	0.011***	0.011***	0.010***	0.011***	0.011***	0.011***
Support	....	....	....	....	1.005	1.005	1.005	1.005
Knowledge	....	....	....	....	1.006	1.006	1.006	1.006
Conflict	....	....	....	....	1.002	1.002	1.002	1.002
Power	....	....	....	....	0.997	0.997	0.997	0.997
Similarity	....	....	....	....	0.993	0.993	0.993	0.993
Status	....	....	....	....	1.008	1.008	1.008	1.008
Trust	....	....	....	....	0.996	0.996	0.996	0.996
Identity	....	....	....	....	1.005	1.005	1.005	1.005
Sentiment Pos.	1.174***	1.173***	1.175***	1.177***	1.174***	1.173***	1.175***	1.177***
Sentiment Neg.	1.080***	1.082***	1.081***	1.081***	1.080***	1.082***	1.081***	1.081***
Comment Left-Wing	....	1.014	....	....	....	1.014	....	....
Comment Right-Wing	....	0.790***	....	....	....	0.790***	....	....
Post Left-Wing	....	0.867***	....	....	....	0.867***	....	....
Post Right-Wing	....	0.852***	....	....	....	0.852***	....	....
Both Polarized	....	....	0.321***	....	....	....	0.321***	....
Both Polarized & Diff. Side	....	....	2.555***	....	....	....	2.555***	....
Diff. Side	....	....	1.022	....	....	....	1.022	....
Shared Group	....	....	....	0.286***	....	....	....	0.286***

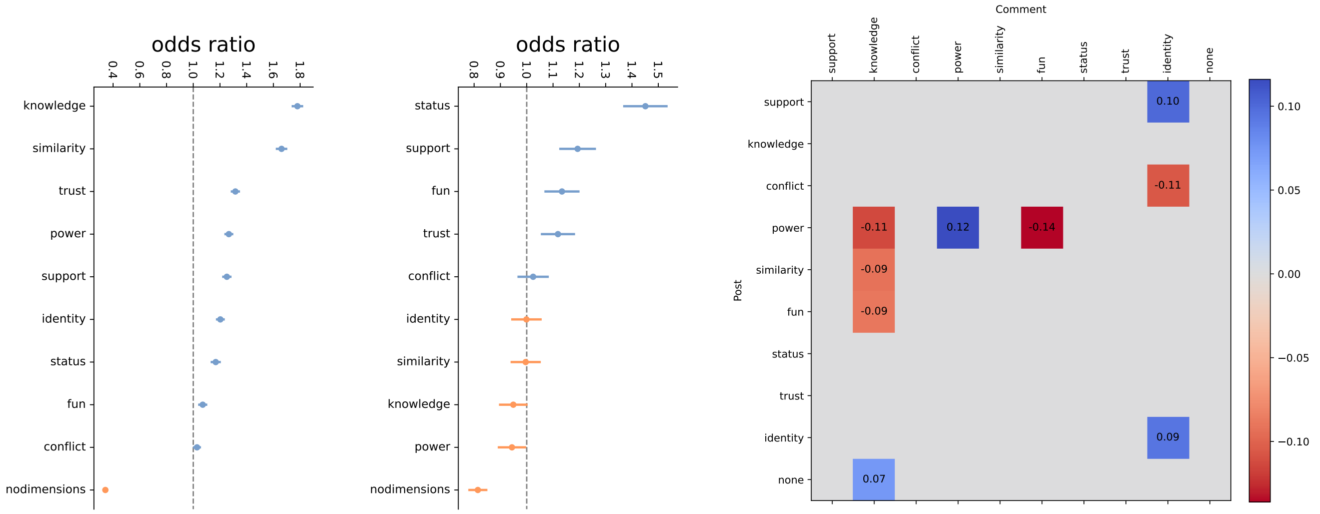

(a) (b) (c)

Figure SI5. Odd ratios of containing a dimension in opinion-changing messages versus the others in the case of (a) comments and (b) posts. Here, we considered only comments with an answer from the original poster. On the right (c), we report only the statistically significant odds ratios ($p < 0.01$) for interactions between dimensions in comments and posts. Results are qualitatively similar to Figure 1.

Table SI8. Odds ratios obtained by logistic regression, analogously to Table 2 in the manuscript, but with a more precise sociopolitical classifier (threshold of 0.75 instead of 0.5).

	A	B	C	D	E	F	G	H
Adj. Pseudo-R^2	0.00285	0.00418	0.00695	0.00925	0.01642	0.01770	0.02043	0.02274
Intercept	0.010***	0.011***	0.010***	0.011***	0.009***	0.010***	0.010***	0.010***
Support	....	....	....	....	1.090***	1.089***	1.091***	1.092***
Knowledge	....	....	....	....	1.214***	1.213***	1.213***	1.213***
Conflict	....	....	....	....	1.030***	1.032***	1.031***	1.031***
Power	....	....	....	....	1.104***	1.106***	1.104***	1.103***
Similarity	....	....	....	....	1.109***	1.107***	1.109***	1.111***
Status	....	....	....	....	0.928***	0.928***	0.933***	0.936***
Trust	....	....	....	....	1.147***	1.147***	1.147***	1.148***
Identity	....	....	....	....	1.091***	1.091***	1.092***	1.092***
Sentiment Pos.	1.170***	1.169***	1.171***	1.173***	1.107***	1.107***	1.107***	1.109***
Sentiment Neg.	1.077***	1.080***	1.079***	1.079***	1.052***	1.054***	1.054***	1.053***
Comment Left-Wing	....	1.012	....	....	....	1.003	....	....
Comment Right-Wing	....	0.785***	....	....	....	0.790***	....	....
Post Left-Wing	....	0.877***	....	....	....	0.883***	....	....
Post Right-Wing	....	0.870***	....	....	....	0.868***	....	....
Both Polarized	....	....	0.336***	....	....	....	0.340***	....
Both Polarized & Diff. Side	....	....	2.372***	....	....	....	2.333***	....
Diff. Side	....	....	1.052***	....	....	....	1.053***	....
Shared Group	....	....	....	0.288***	....	....	....	0.289***

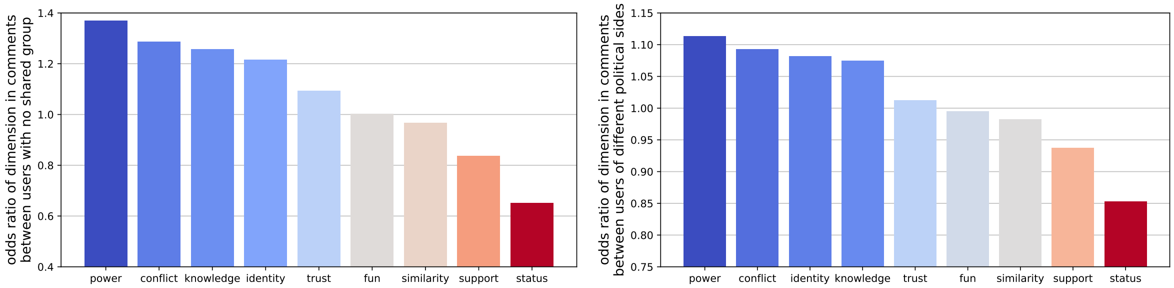

(a) Odd ratios of presence of each dimension in comments between people with (left) no shared subreddits, (right) different political alignments.

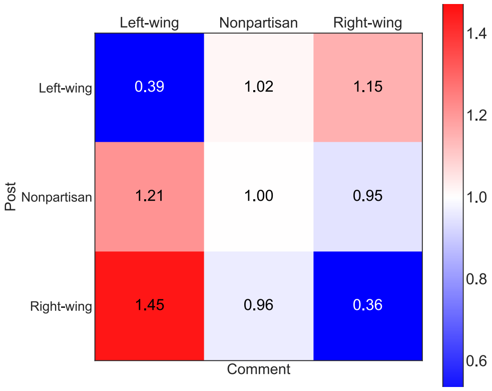

(b) Odds ratio of presence of a Δ in comments between people with a given alignment combination, as estimated by a logistic regression model including these factors and the social dimensions. We normalize the odds ratio with respect to the interaction between two individuals with no detected political side.

Figure SI6. Analysis of political alignment, in relation to (a) social dimensions and (b) opinion change.
